# Case Report: Immunotherapy-containing fertility-sparing treatment integrated with preimplantation genetic testing resulting in live birth in Lynch syndrome-associated endometrial cancer

**DOI:** 10.3389/fonc.2026.1942599

**Published:** 2026-09-11

**Authors:** Yannan Zhao, Yue Zhao, Ting Hu, Yi Guo, Xin Zhang, Shuaishuai Guo

**Affiliations:** 1 Shenyang Clinical Medical Research Center for Assisted Reproduction, Shenyang Women’s and Children’s Hospital, Shenyang, China; 2 Liaoning Key Laboratory of Assisted Reproduction, Shenyang, China; 3 Key Laboratory of Environmental Stress and Chronic Disease Control and Prevention, Ministry of Education, China Medical University, Shenyang, China

**Keywords:** endometrial cancer, fertility-sparing treatment, immune checkpoint inhibitor, Lynch syndrome, PGT-M

## Abstract

**Background:**

Lynch syndrome-associated endometrial cancer (LS-EC) presents unique challenges for fertility preservation. Conventional progestin-based therapy may be less effective in mismatch repair-deficient (MMRd) tumors, whereas immune checkpoint inhibitors (ICIs) have shown promising activity. Meanwhile, preimplantation genetic testing for monogenic disorders (PGT-M) offers a way to prevent hereditary transmission.

**Case presentation:**

A woman was initially diagnosed with complex atypical hyperplasia at age 28 and subsequently developed presumed FIGO 2009 stage IA endometrial cancer (EC) associated with a germline pathogenic variant in *MSH2*. After failing to achieve complete response (CR) following prolonged progestin therapy, she received multimodal fertility-sparing treatment comprising a programmed cell death protein 1 (PD-1) inhibitor (sintilimab 200 mg, 9 cycles) in combination with leuprorelin acetate and a levonorgestrel-releasing intrauterine system. This multimodal regimen achieved CR. After sustained remission was confirmed, PGT-M was performed and transfer of an embryo free of the familial pathogenic variant resulted in a healthy live birth.

**Conclusion:**

This case illustrates the feasibility of integrating immunotherapy-containing fertility-sparing treatment with PGT-M to achieve sustained oncologic control while preventing hereditary transmission in carefully selected patients with LS-EC.

## Introduction

Endometrial cancer (EC) is among the most common gynecologic malignancies, and approximately 3% of cases are attributable to Lynch syndrome (LS) (1), an inherited condition driven by germline mutations in mismatch repair (MMR) genes (2). Women with LS face a substantially elevated lifetime risk of EC (3) and tend to develop the disease younger than women with sporadic tumors (4). As universal MMR testing and molecular classification enter routine practice, LS-associated endometrial cancer (LS-EC) is increasingly diagnosed in women of reproductive age (5).

For young patients who desire future fertility, progestin-based conservative treatment offers an alternative to definitive hysterectomy (6). Managing LS-EC under a fertility-sparing strategy poses distinct challenges. Some retrospective studies have suggested that MMR-deficient (MMRd) tumors may have lower regression rates and higher persistence or recurrence risks, although available evidence remains limited and heterogeneous (7, 8). In addition, a 50% probability of transmitting the pathogenic MMR variant to offspring complicates reproductive decision-making (9). Immune checkpoint inhibitors (ICIs) have shown activity in MMRd EC, including in the fertility-sparing setting, while preimplantation genetic testing for monogenic disorders (PGT-M) offers a strategy to prevent vertical transmission of LS-associated variants (10).

Reports tracing the complete clinical course from multimodal fertility-sparing treatment incorporating immunotherapy, through PGT-M assisted reproduction, to live birth have not been described in women with LS-EC. We describe a woman with LS-EC with a germline pathogenic variant in *MSH2* who achieved CR following multimodal treatment that included immunotherapy, subsequently underwent PGT-M, and delivered a healthy infant. We also review the relevant literature and discuss what this case adds to the management of LS-EC.

## Case presentation

A 31-year-old nulligravid woman presented to our reproductive center for assisted reproduction. She had a history of LS-EC treated with fertility-sparing therapy and immunotherapy. In February 2022, at age 28, she underwent hysteroscopic lesion excision at another hospital after transvaginal ultrasound revealed abnormal endometrial thickening. Histopathology returned as complex atypical endometrial hyperplasia (CAH). Pelvic magnetic resonance imaging showed no myometrial invasion or extrauterine spread. Oral medroxyprogesterone acetate (MPA) was started for fertility preservation. After three months, a follow-up hysteroscopic biopsy in May 2022 showed progression: CAH with focal areas suspicious for carcinoma. On immunohistochemistry, there was loss of MSH2 with retained MLH1, PMS2, and MSH6 expression, wild-type p53 staining, and ER/PR positivity, consistent with an MMRd molecular subtype. Comprehensive genomic profiling confirmed microsatellite instability-high (MSI-H) status. Germline testing identified a heterozygous pathogenic *MSH2* variant (c.2131C>T, p.Arg711Ter), confirming Lynch syndrome. Based on the pathological findings, molecular characteristics, and MRI evidence of superficial myometrial invasion without extrauterine spread, the lesion was managed as LS-EC. The tumor was considered presumed FIGO 2009 stage IA EC. Given the patient’s firm desire to preserve fertility, MPA was continued with close surveillance. A repeat biopsy in September 2022 showed pathological normalization of the endometrium. However, serial biopsies over the following months revealed persistent microscopic residual disease. Sustained CR was never achieved on progestin monotherapy.

In April 2023, owing to incomplete response after approximately 15 months of progestin therapy, the patient was referred to another tertiary center. Hysteroscopic biopsy showed partial response: roughly 80% regression and 20% focal residual tumor. Given the MMRd/MSI-H status, treatment was intensified with sintilimab (200 mg, 9 cycles, April 2023 to February 2024) and gonadotropin-releasing hormone agonist (GnRH-a, leuprorelin acetate, 3.75 mg 1/28D). No immune-related adverse events (irAEs) were observed during the treatment. A levonorgestrel-releasing intrauterine system (LNG-IUS) was inserted in June 2023 following two cycles of sintilimab treatment. Follow-up hysteroscopic biopsies in August 2023 and February 2024 both confirmed CR. Leuprorelin acetate was discontinued in September 2024, and the LNG-IUS was removed in December 2024.

After sustained CR was confirmed, the patient proceeded to *in vitro* fertilization and embryo transfer (IVF-ET) at our hospital. Because of the pathogenic *MSH2* variant and its 50% transmission risk, a combined PGT-M and preimplantation genetic testing for aneuploidy (PGT-A) cycle was designed. Thyroid function and fasting blood glucose were assessed prior to controlled ovarian stimulation and were within normal limits. Controlled ovarian stimulation (COS) using a luteal-phase long GnRH agonist protocol was initiated in March 2025, 13 months after the last sintilimab dose. Baseline ovarian reserve assessment showed an anti-Mullerian hormone level of 5.42 ng/mL and bilateral antral follicle counts exceeding 12. Twenty-eight oocytes were retrieved, yielding eight cleavage-stage embryos. Extended culture produced one day-6 blastocyst suitable for trophectoderm biopsy. Two discarded embryos of insufficient quality were used for linkage analysis. A family-specific haplotype was constructed using polymorphic markers flanking the *MSH2* locus, which successfully identified the maternal risk haplotype. Trophectoderm biopsy was followed by whole-genome amplification and next-generation sequencing for both PGT-M and PGT-A. One euploid embryo was confirmed to be free of the maternal pathogenic *MSH2* variant.

**Figure 1:**
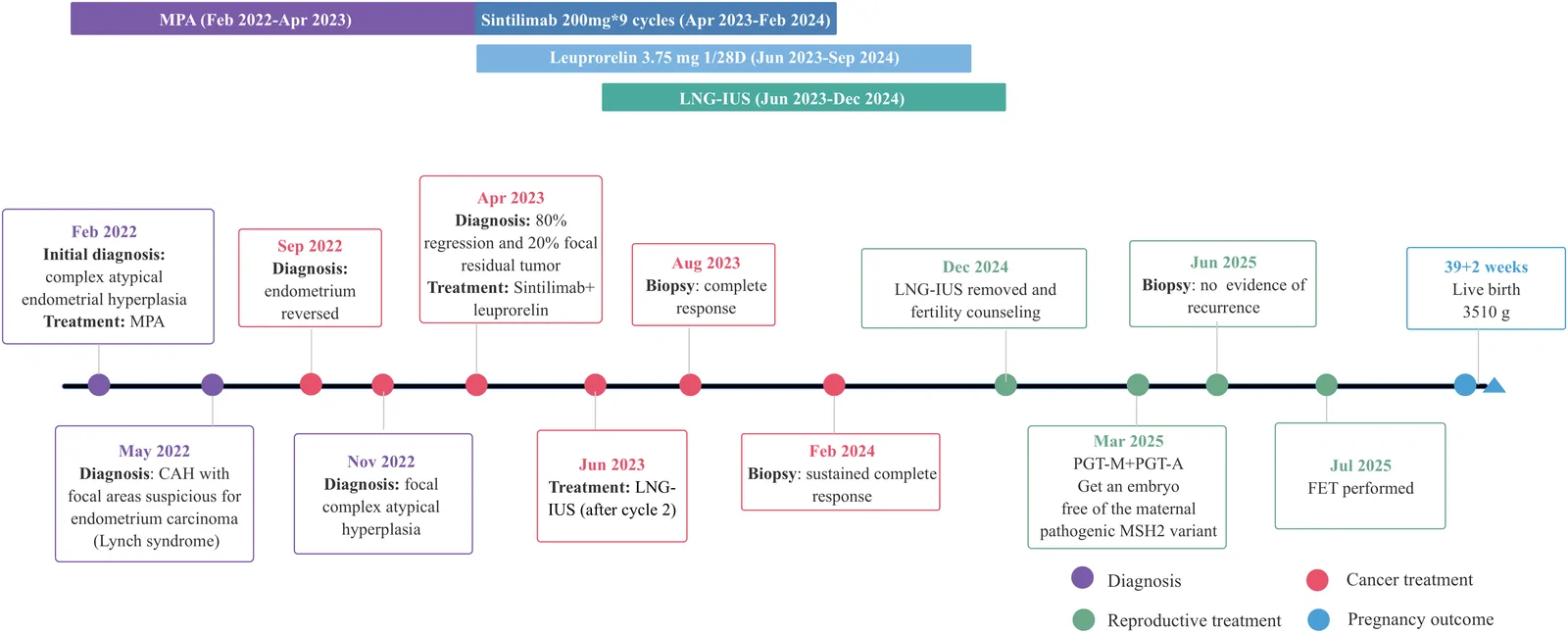
Timeline of the patient's treatment process.

A repeat endometrial biopsy performed prior to embryo transfer showed sustained CR without recurrence. Single frozen-thawed embryo transfer (FET) was then performed, resulting in clinical pregnancy. Prophylactic cervical cerclage was placed at 20 weeks of gestation. The pregnancy progressed uneventfully, and a healthy infant weighing 3510 g was delivered at 39 + 2 weeks of gestation in March 2026. The neonatal Apgar score was 10. Placental histopathological examination showed no abnormal findings. The complete clinical course is summarized in **Figure 1**.

## Discussion

To our knowledge, this is the first report in which fertility‑sparing treatment that included immunotherapy was combined with PGT‑M and resulted in a live birth in a patient with LS‑EC.

LS-EC presents unique challenges for fertility preservation. Affected women are often diagnosed young and frequently desire future childbearing. In this case, prolonged MPA therapy failed to achieve sustained CR despite transient pathological normalization. This fluctuating response is consistent with prior observations that MMRd tumors may respond incompletely to progestin, with recent retrospective studies indicating lower regression rates and higher rates of persistence or recurrence (11–13). The incomplete clearance created the clinical imperative for alternative treatment.

MMRd/MSI-H tumors represent a biologically plausible setting for PD-1 blockade because mismatch repair deficiency may enhance immune recognition. Clinical activity of PD-1 inhibitors has been demonstrated in MMRd/MSI-H EC (14, 15). Sintilimab, a PD-1 inhibitor, has shown clinical activity in advanced EC (16). In this case, sintilimab was administered in combination with hormonal therapy, including a GnRH agonist and LNG-IUS, resulting in CR that was sustained throughout 22 months of follow-up. To contextualize this outcome, we compared the present case with previously published LS-EC patients who received immunotherapy for fertility preservation (**Table 1**). All reported patients achieved CR; however, none underwent PGT-M (17, 18). These findings suggest that immunotherapy-containing regimens may provide a potential option for carefully selected patients with LS-EC who have insufficient response to hormonal therapy. The optimal treatment duration and long-term oncologic safety are not yet known.

**Table 1:** Published cases of fertility-sparing treatment incorporating immune checkpoint inhibitors in Lynch syndrome-associated endometrial cancer.

Reference	Age at diagnosis(yr)	Germline mutation	FIGO Stage (basis)	Other malignancies	Prior Progestin regimen	Concurrent hormonal therapy	ICI regimen	Time to CR	PGT-M	Reproductive outcome	Follow-up after CR	Recurrence
Present case	28	*MSH2* c.2131C>T	presumed FIGO 2009 stage IA	None	MPA 15 mo	Leuprorelin + LNG-IUS	Sintilimab 200 mg; 9 cycles	18 wk	Yes	live birth 39 + 2 wk, 3510 g	22 mo	No
Yang 2025 (17) Case 1	38	*MLH1* 199G>A	IIAm_MMRd_ (clinical)	None	None	3 months megestrol acetate after PD-1 inhibitor	Camrelizumab 200 mg q2w x2 cycles; then pembrolizumab 200 mg q3w x6 cycles	42 wk	No	NR	15.6 mo	No
Yang 2025 (17) Case 2	39	*MLH1* 1975C>T	IA1m_MMRd_ (clinical)	Rectal cancer; ascending colon cancer; synchronous OC	None	No	Pembrolizumab 200 mg q3w x8 cycles	24 wk	No	NR	20.4 mo	No
Yang 2025 (17) Case 3	35	*MSH2* 1864C>T	IA2m_MMRd_ (clinical)	Transverse colon cancer	MA 7 mo; PD; (enantone + letrozole)	No	Sintilimab; 6 cycles	18 wk	No	Live birth; Natural conception; full-term delivery	41 mo	No
Yang 2025 (17) Case 4	40	*MSH2* 1418C>A	IBm_MMRd_ (clinical)	None	LNG-IUS 8 mo; CR; recurrence 13 mo	No	Tislelizumab 200 mg q3w x6 cycles	18 wk	No	NR	3 mo	No
Cao 2022 (18)	36	*MSH2*	presumed clinical stage III	Transverse colon adenocarcinoma (synchronous, T3)	MA 160 mg QD + metformin 7 mo; PD;	No	Sintilimab 200 mg q3w x6 cycles	6 cycles + surgery; pCR (TRG 2)	No	live birth; Natural conception; C-section at 36 wk; male	11 mo	No

CR, complete response; FIGO, International Federation of Gynecology and Obstetrics; ICI, immune checkpoint inhibitor; LNG-IUS, levonorgestrel-releasing intrauterine system; MA, megestrol acetate; MMRd, mismatch repair-deficient; MPA, medroxyprogesterone acetate; NR, not reported; OC, ovarian cancer; PD, progressive disease; PGT-M, preimplantation genetic testing for monogenic disorders; pCR, pathological complete response; QD, once daily; q2w, every 2 weeks; q3w, every 3 weeks; TRG, tumor regression grade; wk, weeks; mo, months.

The optimal fertility-sparing strategy for LS-EC remains undefined. Once CR is achieved, fertility planning and attempts at conception can be considered, with assisted reproductive technology often preferred. PGT-M is a mature technology, but its integration into LS-EC fertility-sparing pathways has rarely been documented. In this case, PGT-M prevented vertical transmission of the pathogenic variant. PGT-M identified an embryo free of the familial pathogenic variant, and concurrent PGT-A confirmed chromosomal euploidy. The FET strategy also provided a critical safety window. A repeat endometrial biopsy performed one month before transfer showed ongoing remission, and the supraphysiologic estradiol exposure from COS was kept separate from the implantation cycle. Although the pre-transfer biopsy was negative in this case, close endometrial surveillance throughout the assisted reproduction process remains prudent.

Although 28 oocytes were retrieved, only one transferable blastocyst was obtained following extended culture. This highlights that adequate oocyte numbers do not guarantee a usable embryo after genetic screening, emphasizing the importance of early fertility counseling. From the patient’s perspective, PGT-M provided reassurance by avoiding hereditary transmission before pregnancy, and she expressed no regret regarding the treatment pathway.

This report has some limitations. First, tumor immune profiling, including PD-L1 expression, tumor mutational burden and lymphocytic infiltration, was not performed. Second, the relative contribution of sintilimab, GnRHa, and LNG-IUS to the observed CR cannot be isolated. Third, long-term neonatal follow-up has not yet been conducted. Fourth, the patient’s oncologic surveillance remains ongoing. Prospective studies are needed to confirm this strategy, define optimal patient selection criteria, and establish standardized monitoring protocols.

## Conclusion

This case shows that CR achieved with multimodal treatment incorporating immunotherapy can be safely bridged to PGT-M and embryo transfer, yielding both successful live birth and prevention of hereditary transmission in a carefully selected patient with LS-EC. Prospective studies are needed to validate its efficacy and long-term safety, define optimal patient selection criteria, and establish standardized protocols for fertility-sparing treatment incorporating immunotherapy in LS-EC.

## Data Availability

The original contributions presented in the study are included in the article/supplementary material. Further inquiries can be directed to the corresponding author.
